# Quantifying research hotspots and trends in brucella spondylitis: a bibliometric analysis

**DOI:** 10.3389/fsurg.2024.1465319

**Published:** 2025-01-07

**Authors:** Zhangui Gu, Zongqiang Yang, Le Fei, Daihao Wei, Long Ma, Qiang Liu, Jiandang Shi

**Affiliations:** ^1^ Department of Orthopedic, General Hospital of Ningxia Medical University, Yinchuan, Ningxia, China; ^2^ First Clinical Medical College of Ningxia Medical University, Yinchuan, Ningxia, China

**Keywords:** brucella spondylitis, bibliometric analysis, management, research trends, spinal infections

## Abstract

**Background:**

Human brucellosis is the most common bacterial zoonosis worldwide, with brucella spondylitis (BS) being one of its most severe forms, potentially leading to spinal deformity or paralysis. This study aims to provide a comprehensive overview of the current status and research trends in the BS field using bibliometric methods.

**Methods:**

Publications on BS from January 1, 1980, to March 24, 2024, were retrieved from the Web of Science database. We used Biblioshiny, VOSviewer, Scimago Graphica, CiteSpace, and Microsoft Office Excel Professional Plus 2016 to analyze publication frequency, geographic distribution, institutional affiliations, international collaborations, authorship, journal sources, keyword usage, trends, and cited references.

**Results:**

Between January 1, 1980, and March 24, 2024, 197 publications on BS were analyzed. Turkey emerged as the leading contributor, with 62 publications, accounting for 31.47%. Weibin Sheng was the most prolific author, contributing 7 papers (3.55%). Xinjiang Medical University was the leading institution with 13 documents (6.60%). *Medicine* and *Rheumatology International* each published 6 papers (3.05%). CiteSpace analysis highlighted “spinal brucellosis,” “spondylitis,” “complications,” “diagnosis,” and “involvement” as the core research areas in BS. Keyword clustering analysis identified 11 primary clusters representing the main research directions. Analysis of abstracts and keyword trends revealed that post-2020, emerging research frontiers include “instrumentation,” “management,” and “debridement.”

**Conclusion:**

There has been significant progress in BS research, with a steady increase in publications. Current research focuses on diagnosis and complications, while future studies may explore management and instrumentation. Increased collaboration among countries and researchers is recommended.

## 1 Introduction

Human brucellosis (HB) is a widespread zoonotic infection caused by various species of the *Brucella* genus. Recent research indicates a significant global rise in HB incidence, with annual new cases estimated between 1.6 and 2.1 million (1, 2). Recognized as the most common bacterial zoonosis worldwide, HB remains a significant public health challenge (3), particularly in regions like the Mediterranean, India, Latin America, the Middle East, and numerous African countries north and south of the Sahara Desert (4). The disease is known for its chronic and recurrent nature, affecting multiple systems and organs, with brucella spondylitis (BS) being one of its most severe manifestations (5). The prevalence of spondylitis in brucellosis cases varies widely, ranging from 2% to 60% (6).

The clinical symptoms of BS are varied, including chronic progressive back pain, localized swelling, restricted movement, and fever. The wide range of symptoms and the absence of specific indicators often delay diagnosis, leading to severe complications such as spinal instability, spinal cord compression, epidural mass formation, or paralysis (7). Therefore, timely diagnosis is crucial for effective management and prognosis. Traditional microbiological and serological agglutination tests assist in diagnosing BS but have poor sensitivity (8). Early imaging is often indistinguishable from other forms of infectious spondylitis, such as spinal tuberculosis (STB), making early diagnosis challenging (9). Laboratory tests, including hemoglobin level, platelet count, white blood cell count, erythrocyte sedimentation rate (ESR), and C-reactive protein (CRP), are essential for assessing treatment efficacy and predicting prognosis. However, these tests do not offer diagnostic specificity for BS (10, 11). Recently, nucleic acid amplification tests (NAATs) and molecular biology techniques have shown promise as early diagnostic tools, though extensive clinical studies are needed to confirm their accuracy and reliability (12, 13).

Conservative management is the most commonly reported treatment for BS (14). Early diagnosis, strict adherence to antibiotic regimens, and regular follow-ups contribute to successful management (15). However, relapse and sequelae remain significant concerns, and the optimal antibiotic regimen and course are still controversial (16). Surgery aims to restore spinal stability, relieve spinal cord or nerve compression, and remove diseased tissue. This includes filling defective areas, excising and draining abscesses, and performing biopsies for histopathological examination and culture to provide early diagnosis and treatment guidance (17). Unfortunately, there are no standardized indications for surgical intervention, which remains controversial (18, 19).

Bibliometrics is an invaluable scientific tool for analyzing research within a field. Using software such as Biblioshiny, VOSviewer, and CiteSpace, bibliometrics systematically processes publication data and precisely visualizes research content. This helps identify research focal points quickly and predict outcomes, guiding scholars toward specific research directions. By integrating and processing bibliometric information, comprehensive discussions within a field become more scientific and rigorous. Consequently, bibliometrics has gained increasing attention and application in recent years. This paper aims to provide a comprehensive analysis and summary of publications related to BS using bibliometric methods and to explore the research hotspots and frontiers of BS.

## 2 Materials and methods

### 2.1 Sources of data

This study utilized the Web of Science Core Collection (WoSCC) database, known for its comprehensive citation and publication data across various scientific disciplines.

### 2.2 Search strategies

**Figure 1:**
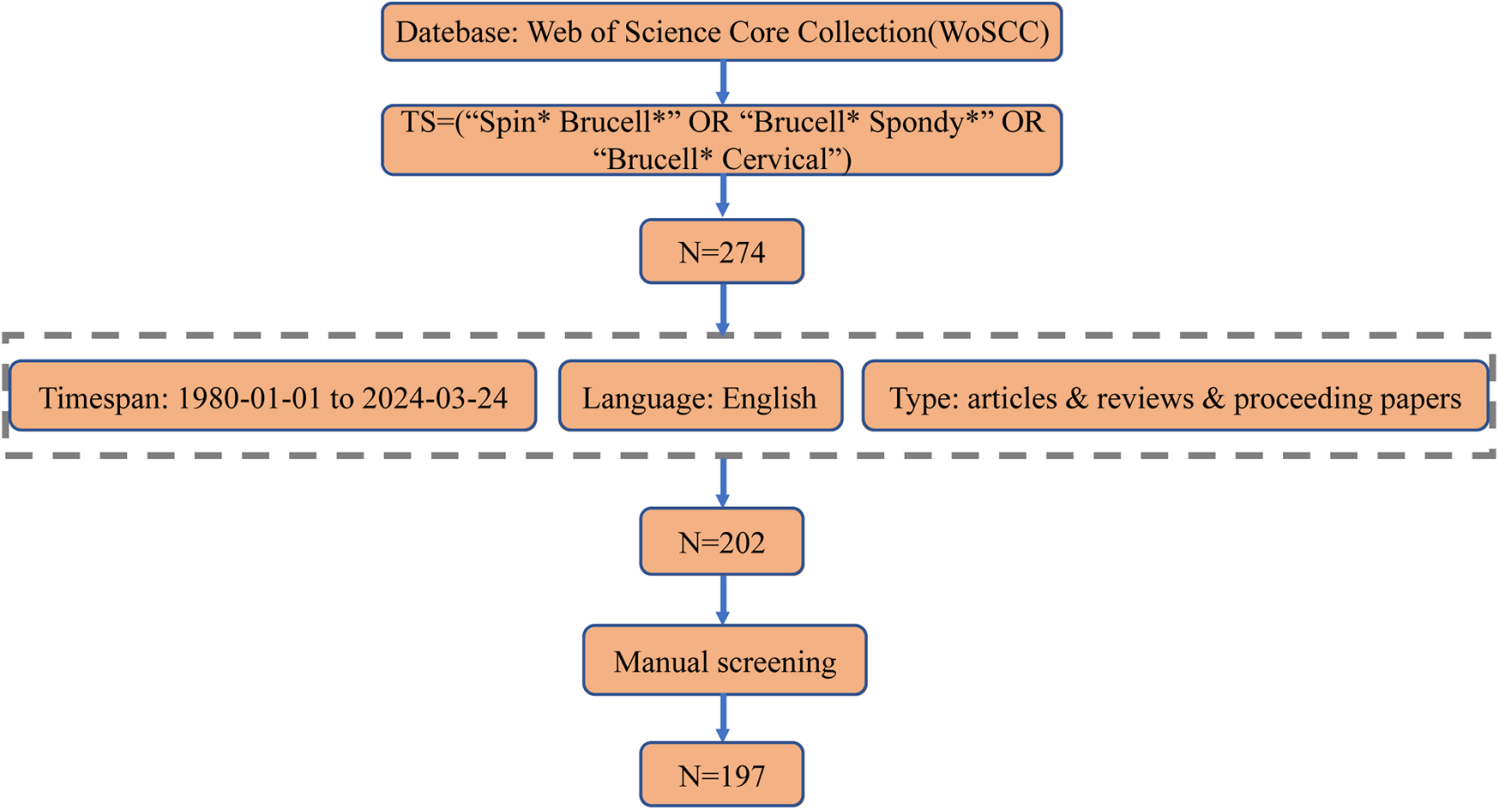
Search strategy framework flowchart.

Two investigators searched WoSCC on March 24, 2024, to identify relevant studies published from January 1, 1980, to March 24, 2024. The search terms were as follows: TS = (“Spin* Brucell*” OR “Brucell* Spondy*” OR “Brucell* Cervical”) AND publishing year = (1980–2024) AND document types = (articles & reviews & proceedings papers) AND language = (English). This search yielded 274 articles. The following categories were excluded: editorial material, letters, book chapters, meeting abstracts, early access, retractions, retracted publications, and corrections. A manual review was conducted to ensure the selected publications were directly relevant to BS research. This review involved two authors (Zhangui Gu and Zongqiang Yang), with any discrepancies resolved by the experienced corresponding author (Jiandang Shi). The search results were exported as a plain text file, with the record content comprising the “Full Record and Cited References.” The final analysis included 197 publications. The retrieval strategy employed in the study is illustrated in Figure 1.

### 2.3 Bibliometric analysis

The bibliometric analysis was conducted systematically, beginning with an overview of publications and countries/regions using Microsoft Office Excel Professional Plus 2016. The data were then imported into Biblioshiny (R version 4.3.3), CiteSpace (6.3.R1), and VOSviewer (1.6.20) for further analysis, including institutions, authors, journals, document citations, reference co-citations, keywords, and trends. The country collaboration map was created using Scimago Graphica (1.0.41).

Biblioshiny evaluates several bibliometric indicators to assess the output of authors, institutions, and journals. The number of articles measures productivity, total citations indicate the impact on the scientific community, and local citations assess the impact in specific fields. These dimensions are used to evaluate research quality. The h-index, which combines productivity and effect, indicates that a researcher has published h papers, each cited at least h times. Additionally, Biblioshiny plotted a three-field plot and authors’ production over time. Data exported from VOSviewer were used in Scimago Graphica to generate a geographic distribution map of BS publications (20). In the VOSviewer map, nodes represent items such as countries, organizations, and authors, with node size indicating the number of these items. Lines between nodes reflect the degree of cooperation or citation among projects, indicated by the same color (21). CiteSpace, a Java application for bibliometric analysis developed by Chen, supports knowledge mining and data visualization (22). It generated bibliographic citations, reference co-citations, keyword co-occurrences, keyword clusters, and timeline plots and analyzed keyword bursts representing trending themes over time. Microsoft Office Excel Plus 2016 was used to create annual production and average citation graphs.

## 3 Results

### 3.1 Search results

**Figure 2:**
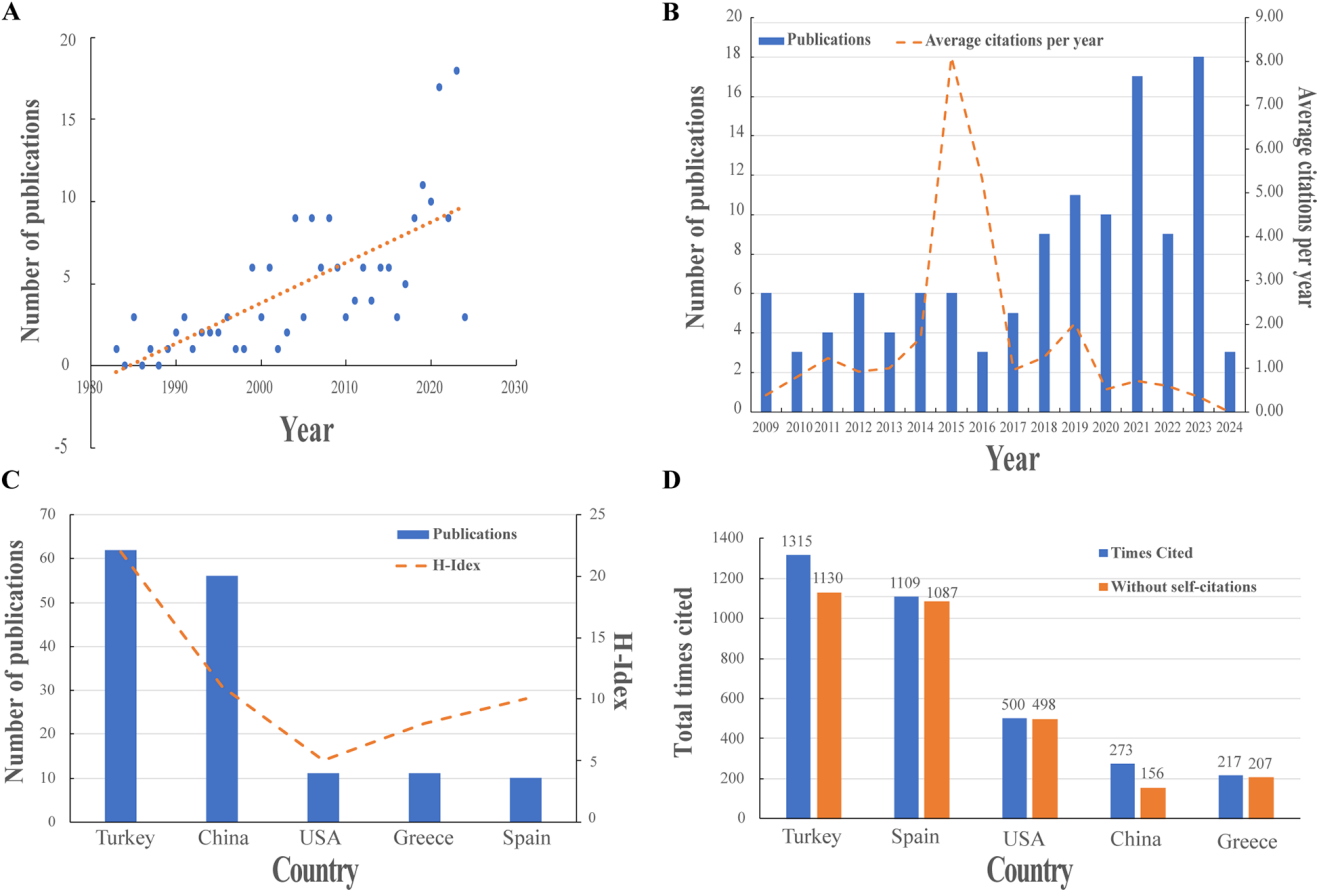
Visualization of publications. **(A)** The trend of BS publications. **(B)** The number of BS publications from 2009 to 2024. **(C)** Top five countries’ number of publications and H-index. **(D)** The top five countries in terms of citations.

The study included 197 publications, comprising 178 articles, 15 reviews, and 4 proceeding papers (Figure 1). The number of publications on BS has increased significantly, from 6 in 1999 to a peak of 18 in 2023 (Figure 2A). Figure 2B illustrates the trend in the number of BS-related publications and the average number of citations per year from 2009 to 2024.

### 3.2 Countries and institutions

**Figure 3:**
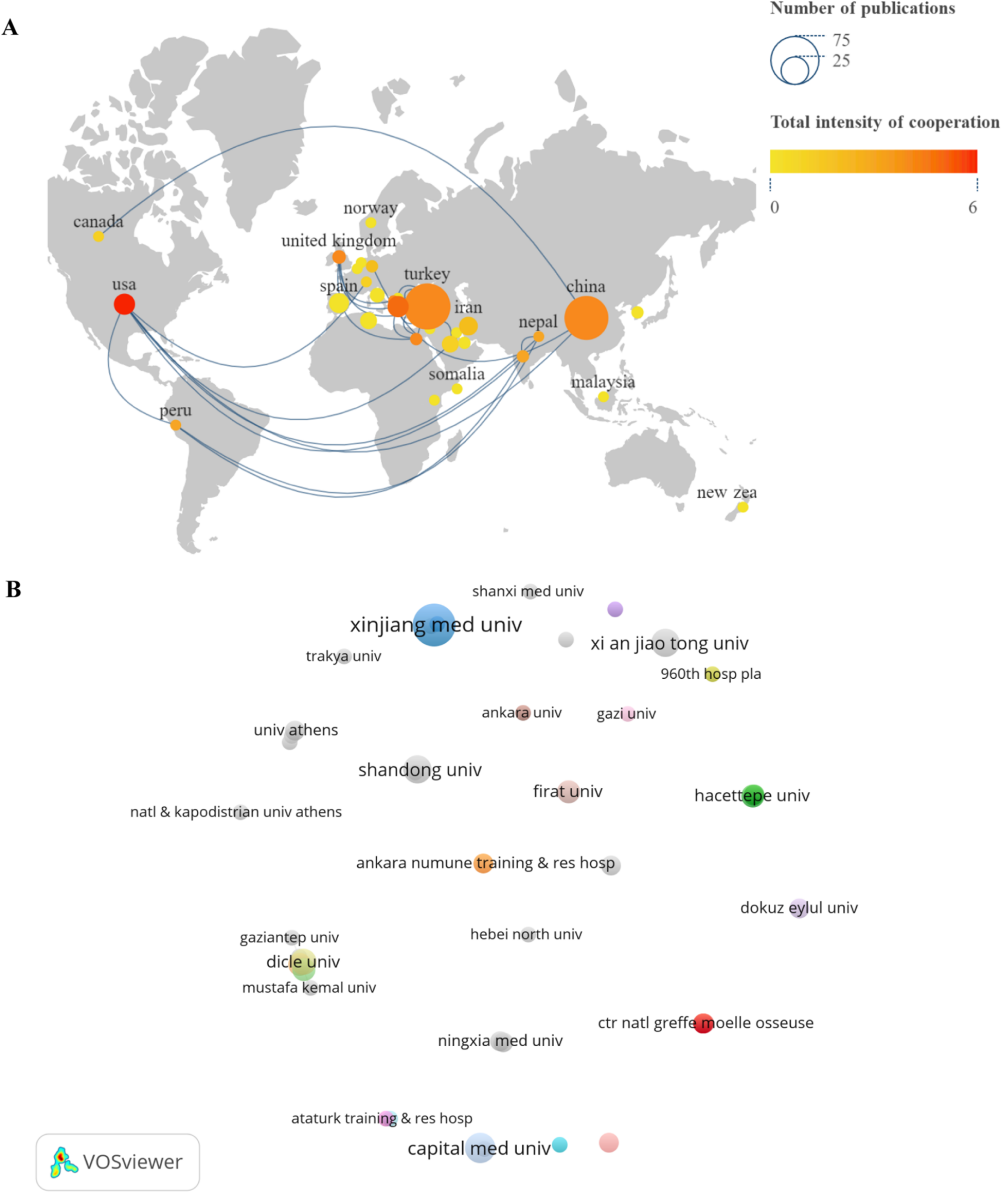
Visualize and analyze active countries and institutions. **(A)** Country cooperation map. **(B)** A collaborative network of institutions.

Researchers from 33 countries contributed to BS research, with Turkey leading in the number of publications (62, 31.47%), followed by China (56, 28.43%), the USA (11, 5.58%), Greece (11, 5.58%), and Spain (10, 5.08%) (Figure 2C). Table 1 lists the top 10 countries in terms of publications. Turkey also ranks first in citation count with 1315 citations, followed by Spain (1109) and the USA (500) (Figure 2D), highlighting Turkey's significant influence in this field. Figure 3A presents a map showing the number of publications and cooperation strength between countries, with the USA initiating and participating in the most collaborations, though generally with fewer partnerships involving other countries.

**Table 1:** The Top 10 countries and institutions in BS research.

Rank	Country/Region	Count (%)	Institution	Count (%)
1	Turkey	62 (31.47)	Xinjiang Medical University	13 (6.60)
2	China	56 (28.43)	Capital Medical University	7 (3.55)
3	Greece	11 (5.58)	Shandong University	6 (3.05)
4	USA	11 (5.58)	Xi'an Jiaotong University	6 (3.05)
5	Spain	10 (5.08)	Dicle University	5 (2.54)
6	Saudi Arabia	9 (4.57)	Firat University	4 (2.03)
7	Iran	8 (4.06)	Hacettepe University	4 (2.03)
8	Tunisia	6 (3.05)	Erciyes University	4 (2.03)
9	Italy	4 (2.03)	Baskent University	4 (2.03)
10	England	4 (2.03)	Ningxia Medical University	3(1.52)

A total of 269 institutions produced the 197 publications. Among the top 10 institutions (Table 1), Xinjiang Medical University published the highest number of articles (13, 6.60%), followed by Capital Medical University (7, 3.55%), Shandong University (6, 3.05%), Xi'an Jiaotong University (6, 3.05%), and Dicle University (5, 2.54%). Figure 3B illustrates the collaboration between these institutions, with a network map highlighting the 50 institutions that published at least two articles. The size of the circles in the map represents the number of publications and the extent of collaboration within the same cluster.

### 3.3 Authors and journal distribution

**Figure 4:**
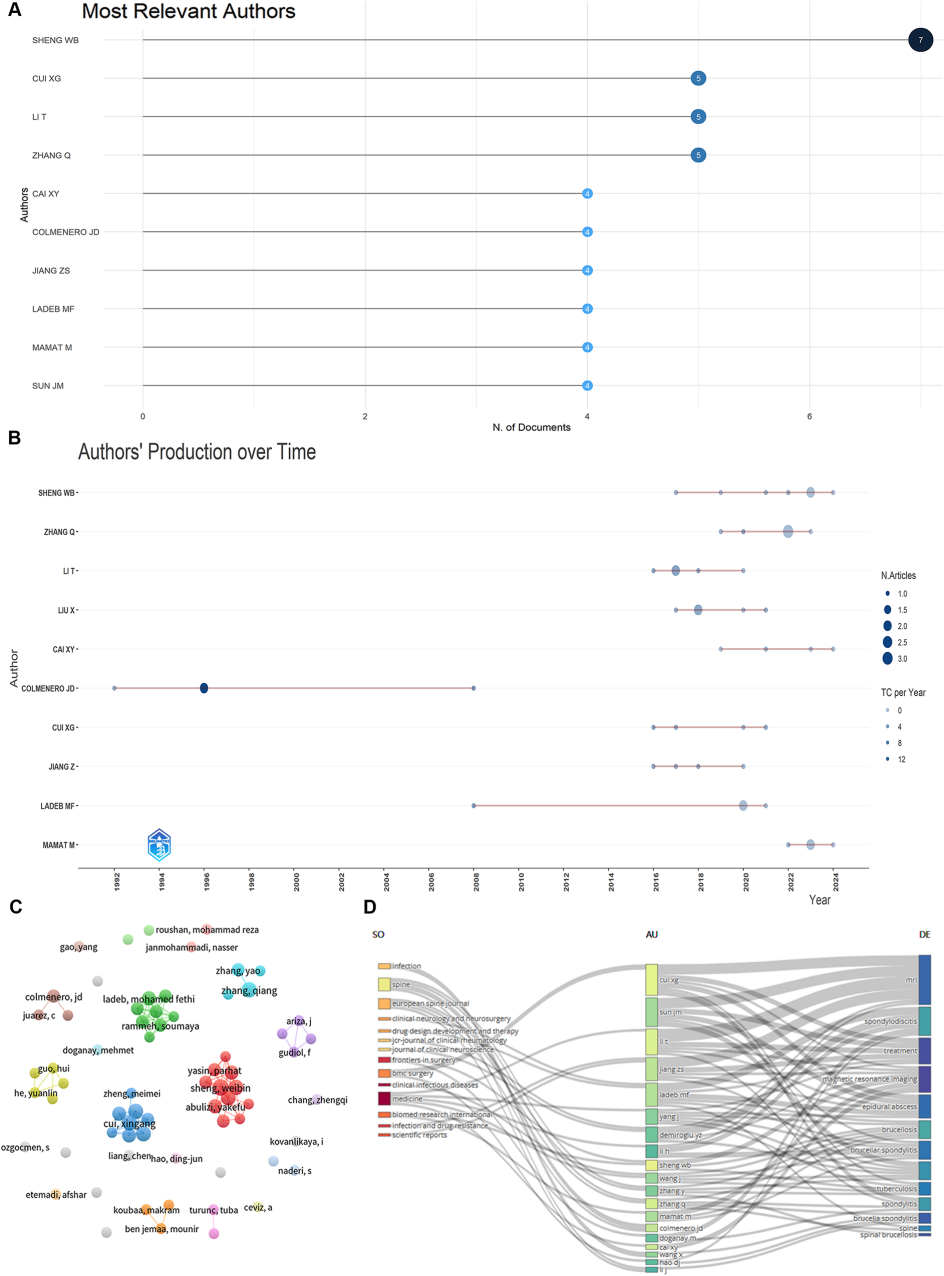
Visualize and analyze active authors. **(A)** Most relevant authors. **(B)** Authors’ production over time (The nodes’ size and darkness are proportional to the number of documents the connection produces). **(C)** The network map of authors. **(D)** Three-field plot (Middle field: Authors, left field: Sources, right field: Keywords).

A total of 912 authors contributed to BS research. The top 10 authors, listed in Figure 4A, accounted for 46 publications (23.35%). Sheng WB was the most prolific, with 7 publications (3.55%), followed by Li T (5, 2.54%) and Cui XG (5, 2.54%). Figure 4B shows the publication years of the top 10 authors. Colmenero JD began his research on BS in 1992. Using the H-index and G-index for bibliometric assessment, Cui XG, Li T, and Colmenero JD emerged as the top three scholars in the field (Table 2). Figure 4C displays a network graph of authors with at least two publications, comprising 68 nodes and 13 clusters.

**Table 2:** H-index and G-index ranking of the Top 10 authors.

Rank	Author	H-index	Author	G-index
1	Colmenero JD	4	Cui XG	5
2	Cui XG	4	Li T	5
3	Li T	4	Colmenero JD	4
4	Demiroglu YZ	3	Jiang ZS	4
5	Doganay M	3	Sheng WB	4
6	Jiang ZS	3	Sun JM	4
7	Li H	3	Ladeb MF	4
8	Ozgocmen S	3	Zhang Q	4
9	Sheng WB	3	Demiroglu YZ	3
10	Sipsas NV	3	Doganay M	3

**Figure 5:**
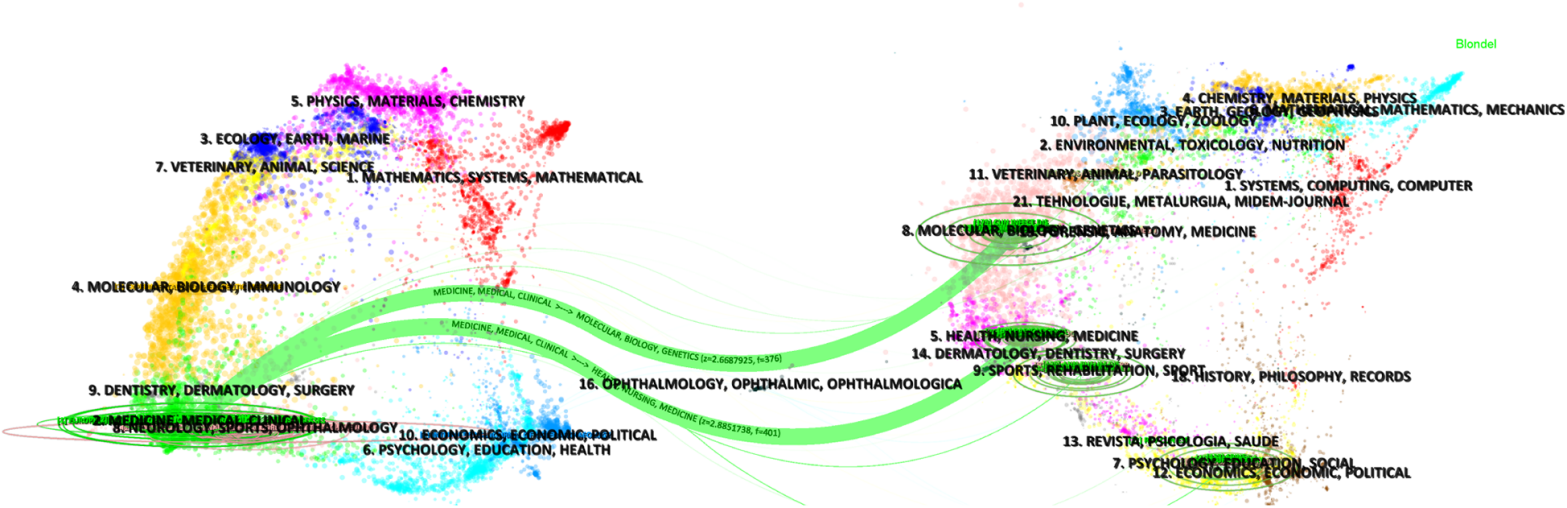
The dual-map overlay of journals related to BS.

BS literature has been published in 130 academic journals, with the top 10 journals contributing 40 publications (20.30%) (Table 3). *Rheumatology International* and *Medicine* lead with 6 publications each (3.05%), followed by *Spinal Cord* (5, 2.54%) and *Clinical Infectious Diseases* (4, 2.03%). Figure 4D visualizes the distribution of authors across different topics and journals. The main research keywords among the top 20 authors include magnetic resonance imaging, spondylodiscitis, treatment, epidural abscess, brucellosis, and brucella spondylitis, with their work typically published in journals like *Spine*, *European Spine Journal*, and *Medicine*. A dual-map overlay of journals (Figure 5) shows BS article publications on the left and cited journals on the right, highlighting the topics covered by cited journals.

**Table 3:** The Top 10 journals in BS research.

Rank	Journal	Publications	Total citations	IF (2022)	H-index
1	Rheumatology International	6	136	4.00	6
2	Medicine	6	484	1.60	4
3	Spinal Cord	5	85	2.20	5
4	Clinical Infectious Diseases	4	729	2.50	4
5	Cureus Journal of Medical Science	4	1	1.20	1
6	Clinical Imaging	3	98	0.67	3
7	Clinical Microbiology and Infection	3	92	2.35	3
8	Clinical Rheumatology	3	21	3.10	3
9	Spine Journal	3	68	4.60	3
10	Journal Of Infection in Developing Countries	3	21	1.70	2

### 3.4 Citation of documents and co-citation of references

**Figure 6:**
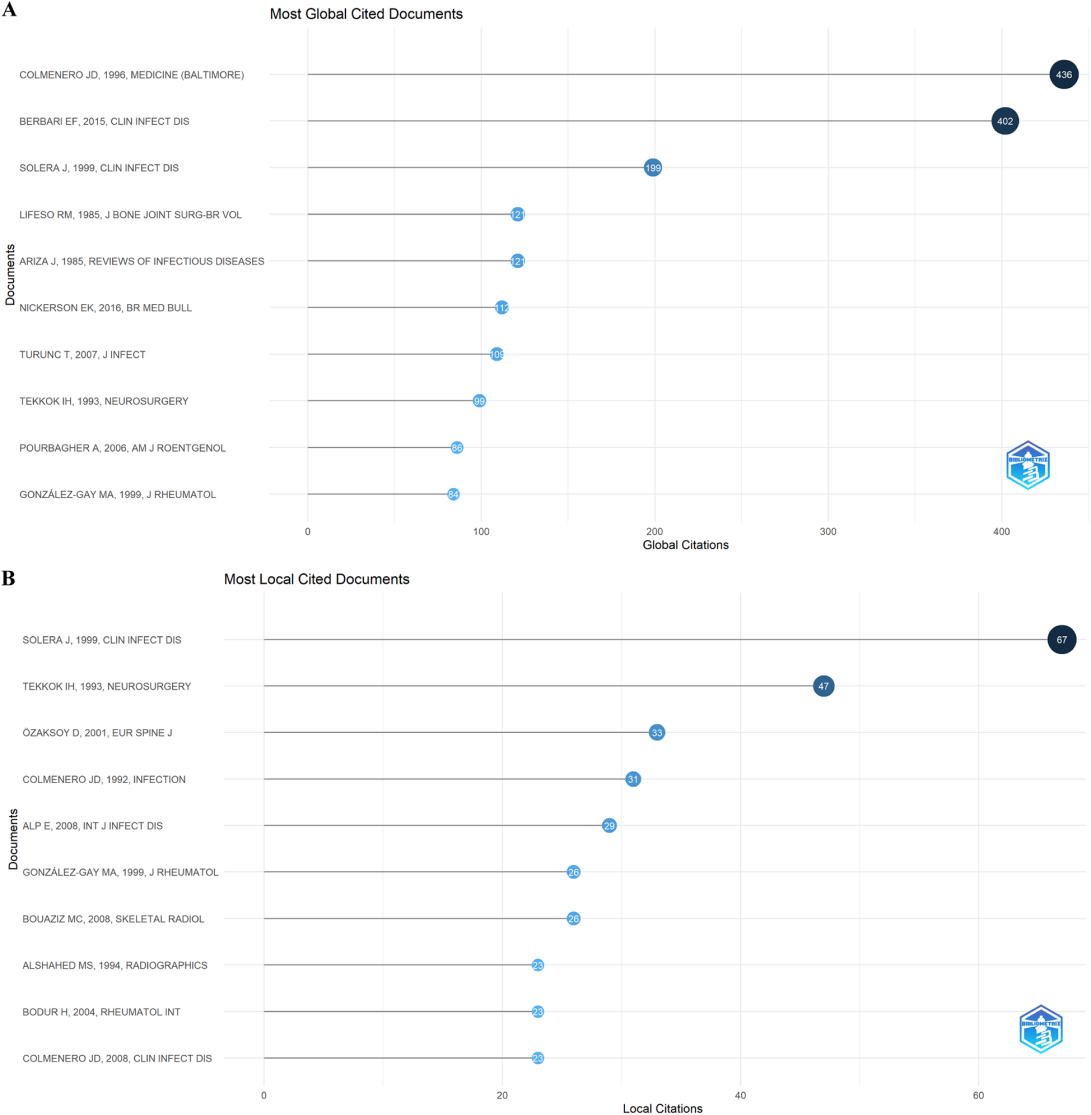
Visualize and analyze documents analysis. **(A)** Most global cited documents. **(B)** Most local cited documents.

Based on WoSCC citation data, the top 10 cited papers globally and locally are shown in Figures 6A,B. The top three globally cited articles had 436, 402, and 199 citations, authored by Colmenero JD, Berbari EF, and Solera J, respectively. The top locally cited paper, by Javier Solera, focuses on BS diagnosis and treatment.

**Figure 7:**
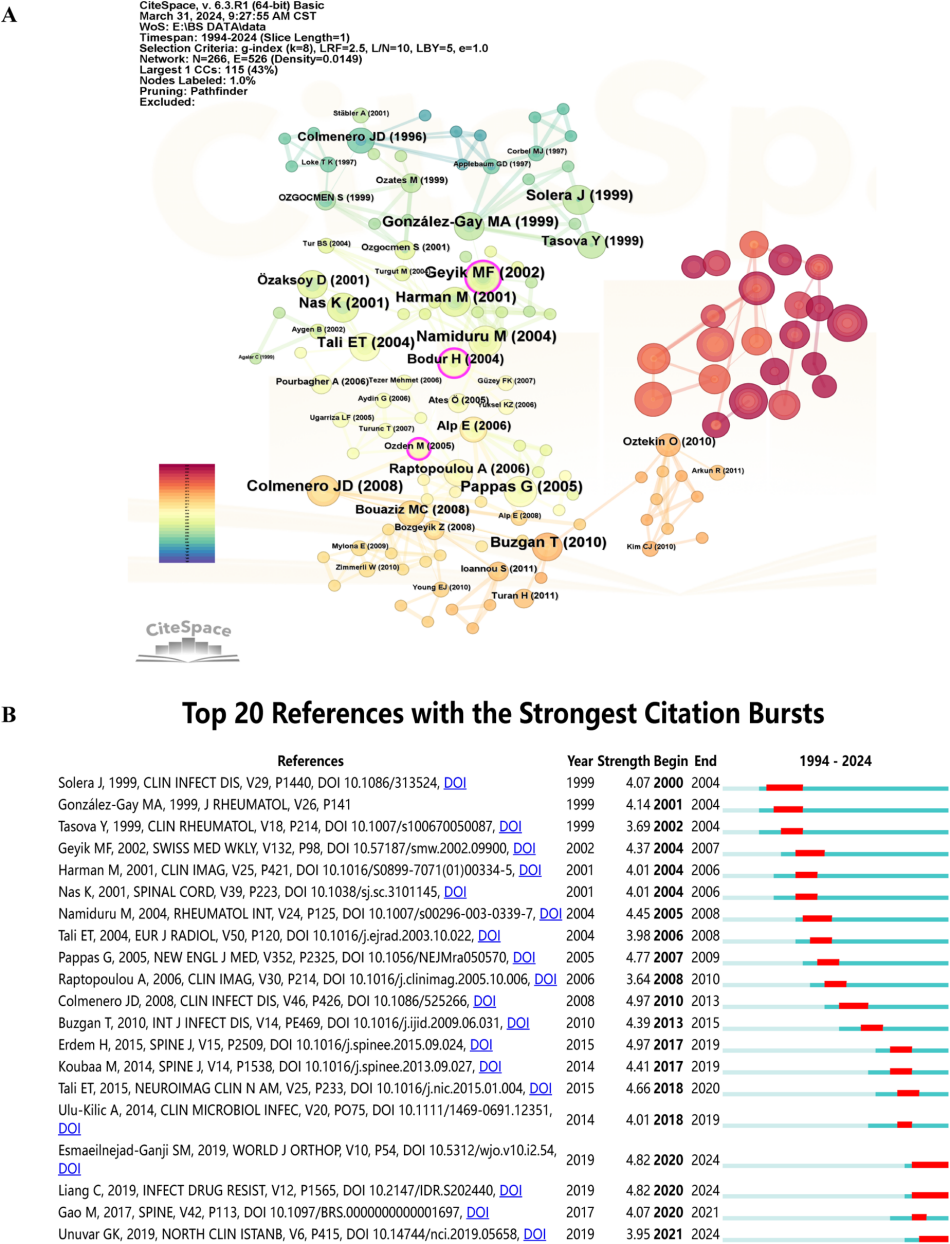
Visualize and analyze references analysis. **(A)** Cluster analysis of co-cited references. **(B)** Top 20 references with the strongest citation bursts.

Co-cited references are depicted as nodes in Figure 7A, with the top three in terms of centrality intensity being “Brucellar spondylitis,” “Musculoskeletal involvement of brucellosis in different age groups: a study of 195 cases,” and “A case of brucella spondylodiscitis with extended, multiple-level involvement.” Figure 7B shows the top 20 references with the strongest citation bursts, highlighting their citation burst intensity and duration.

### 3.5 Keywords and trends

**Figure 8:**
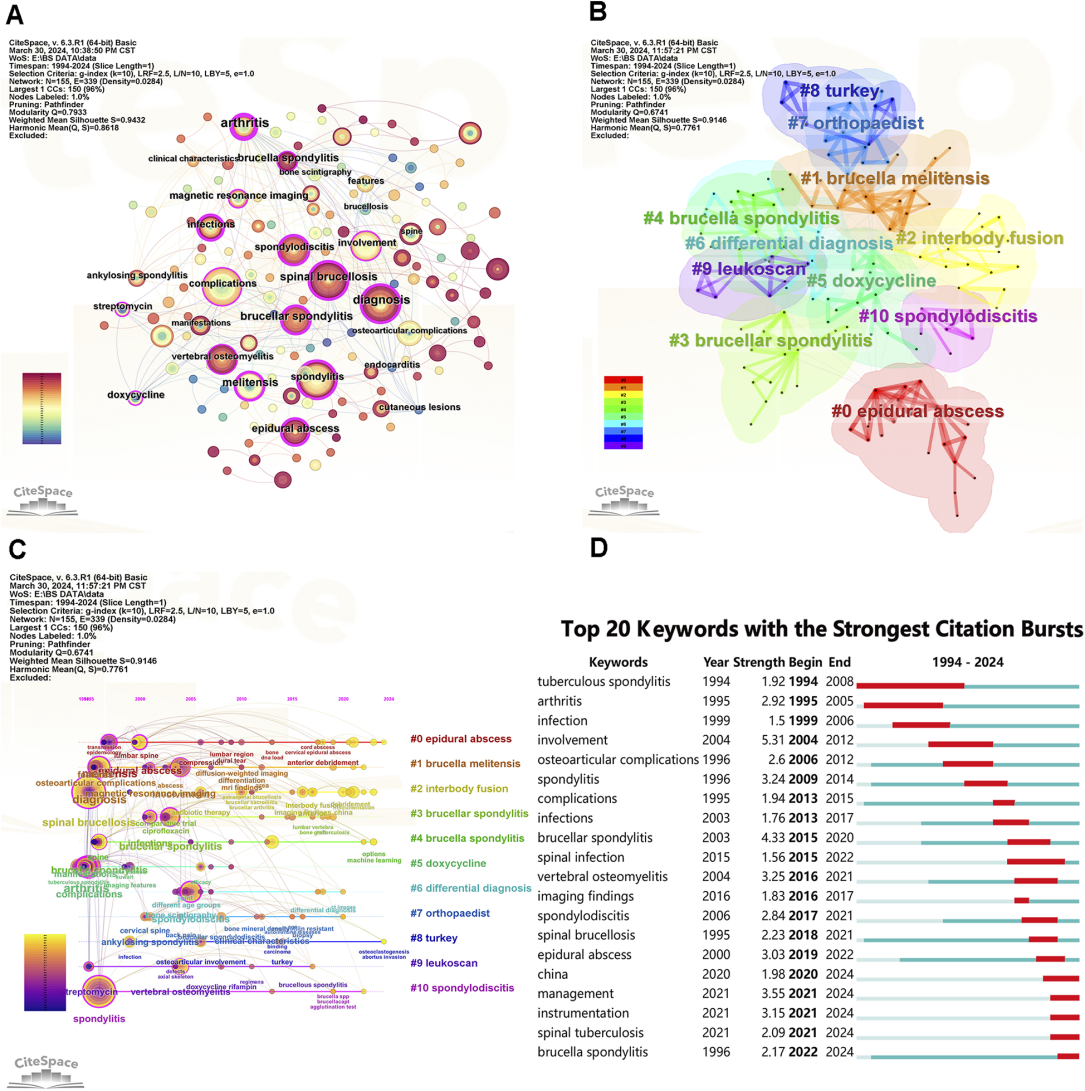
Visualize and analyze keywords analysis. **(A)** The network map of keywords. **(B)** Clusters of keywords. **(C)** Keyword timeline view in BS research. **(D)** Top 20 keywords with the strongest citation bursts.

Figure 8A presents a high-frequency network map of keywords generated by CiteSpace. The keywords “spinal brucellosis” and “spondylitis” were predominant, along with frequently used terms like “complications,” “diagnosis,” and “involvement.”

Keyword clustering in Figure 8B reveals 11 significant research directions, including “epidural abscess,” “brucella melitensis,” “interbody fusion,” “brucellar spondylitis,” “brucella spondylitis,” “doxycycline,” “differential diagnosis,” “orthopaedist,” “turkey,” “leukoscan,” and “spondylodiscitis.”

The timeline view of keywords in Figure 8C shows that BS research primarily focuses on clinical studies, such as clinical features, diagnosis, imaging presentations, and complications. In recent years, areas like essential research and machine learning have emerged.

**Figure 9:**
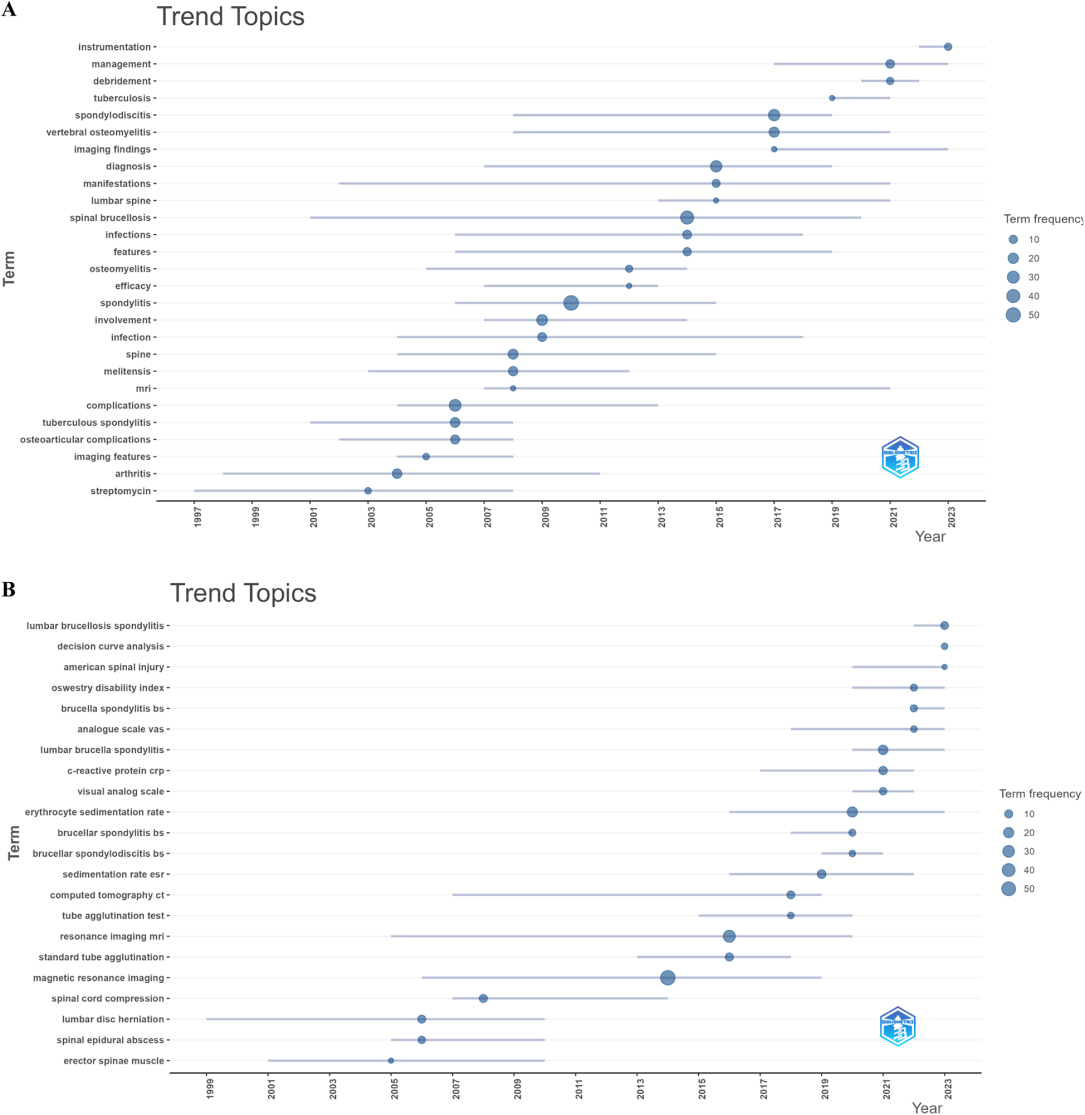
Visualize and analyze trend topics. **(A)** Abstracts (trigram). **(B)** Keywords plus (Circle size represents frequency).).

Keyword burst detection analysis reveals significant research trends and hotspots by identifying notable changes in keyword occurrence frequency (Figure 8D). Since 2020, the keywords with the strongest citation bursts are “management” (2021–2024), “instrumentation” (2021–2024), “spinal tuberculosis” (2021–2024), and “brucella spondylitis” (2022–2024). Trend Topics in Biblioshiny, shown in Figures 9A,B, further clarify abstract and keyword topic trends. These analyses indicate that “instrumentation,” “management,” and “debridement” have emerged as crucial research frontiers post-2020.

## 4 Discussion

### 4.1 Overview of *Brucella* spondylitis

BS is an acute or chronic spinal infection caused by *Brucella* species. Key clinical manifestations include fever, persistent back pain, neurological symptoms, and systemic signs. In the early stages of the disease, the clinical presentation of BS closely resembles that of spinal tuberculosis and pyogenic spondylitis, and differentiating these conditions based on laboratory tests, imaging, and pathology is challenging, further complicating the diagnosis (23). Misdiagnosis or delayed diagnosis can result in severe complications such as spinal deformities, neurological impairment, or even permanent disability (24). Therefore, timely and accurate diagnosis and intervention are critical to prevent serious sequelae and to alleviate the economic burden on patients and society.

The treatment of BS is typically tailored according to the location and severity of the infection. Combination antibiotic therapy remains the cornerstone of treatment, with common regimens involving prolonged courses of antibiotics such as doxycycline and rifampicin (19). For patients experiencing neurological deficits or spinal instability, surgical intervention is often necessary. Common surgical procedures include debridement, interbody fusion, and mechanical stabilization using internal fixation devices (25). Minimally invasive surgical techniques have gained popularity due to their reduced trauma and faster recovery, making them particularly beneficial for patients with early-stage disease or mild neural compression. However, the choice of surgical approach should be based on the anatomical site of the lesion and the severity of the condition.

In recent years, significant advancements have been made in understanding the pathogenesis and optimizing treatment strategies for BS. This study utilizes a bibliometric analysis of literature from the WoSCC database, spanning from 1980 to 2024, to elucidate the current research landscape, emerging trends, and key research topics in the field. The insights gained from this analysis offer clear directions for future research on BS, facilitating critical advancements in both basic research and clinical management of the disease.

### 4.2 General information

While the number of publications on BS remains lower than for other spinal infectious diseases like STB (26), there has been a significant increase in BS research from 1980 to 2024. Among the top five countries for BS publications, Turkey, Spain, and Greece, all in the Mediterranean region, contribute significantly, highlighting the higher prevalence of brucellosis in this area (4). Turkey leads with the highest number of papers (62, 31.47%) and total citations (1,315), likely due to a higher HB prevalence (27). China ranks second with 56 publications (28.43%) but has relatively low citations, indicating a need for improved research quality as HB has been re-emerging in China since the 1990s (28). Chinese research on BS began to surge in 2016, with Xinjiang Medical University and scholar Sheng WB making notable contributions. This aligns with earlier reports of high HB incidence in Chinese provinces like Ningxia, Xinjiang, and Inner Mongolia from 2016 to 2018 (29). In contrast, the incidence of HB in the United States dramatically declined during the 2000s (30), and BS cases are now rare (31). However, the U.S. has developed several influential clinical guidelines for diagnosing and treating BS (32). It plays a central role in international cooperation, particularly with Peru, Switzerland, India, Nepal, Iran, and China.

Among the top publishing institutions, five in China and four in Turkey have made significant contributions. However, the cooperation network diagram (Figure 3B) indicates that inter-institutional collaboration needs strengthening. Publications on BS are frequently found in *Rheumatology International* and *Medicine*, followed by *Spinal Cord* and *Clinical Infectious Diseases*. Influential studies by scholars like Bodur H, Ozden M, and Geyik MF form the foundational knowledge base of BS, providing critical insights into its clinical features and treatment. Citation analysis shows that works by Colmenero JD and Erdem H have a significant impact, with recent studies by Liang C and Esmaeilnejad-Ganji SM continuing to attract attention (33–39).

### 4.3 Hotspots and frontiers

The high-frequency co-occurrence of specific keywords highlights research hotspots in BS. The top five keywords are “Spinal brucellosis,” “Spondylitis,” “Complications,” “Diagnosis,” and “Involvement.” These terms dominate BS research, indicating key study areas (Figure 8A). “Involvement” and “complications” in BS research refer to osteoarticular involvement, a common brucellosis complication, and complications from BS progression, such as systemic symptoms, localized muscle spasms, radicular pain due to abscess compression, and spinal cord involvement leading to sensory or motor impairment or restricted spinal motion. medication-induced adverse drug reactions and surgical complications are also significant (40, 41). Due to the non-specific nature of early clinical signs, imaging manifestations, slow blood bacterial culture growth, and complex serodiagnosis, BS remains challenging to differentiate from atypical spinal metastases, STB, and other infectious diseases (42). The keyword “diagnostics” highlights the need for breakthroughs in recognizing these hard-to-distinguish diseases and serious complications. Many studies have explored clinical features, laboratory tests, microbiological tests, imaging, and machine learning for diagnostic advancements (8, 11, 19, 43–49).

The keyword “tuberculous spondylitis” had a burst from 1994 to 2008 and re-emerged as “spinal tuberculosis” from 2021 to 2024 (Figure 8D). This resurgence may reflect early diagnostic and therapeutic confusion between BS and STB, with recent advances in imaging and machine learning offering a deeper understanding of both diseases. Abstract analysis using the trigrams algorithm indicates a focus on clinical studies, with fewer basic studies (Figure 9A). Keyword co-occurrence, indicating a sudden increase in keyword citations, measures a keyword's influence and importance, reflecting research frontiers at specific times. Figures 8D, 9B show that “Instrumentation,” “Management,” and “Debridement” are research frontiers post-2020. Beyond bibliometric software predictions, machine learning could emerge as a research frontier after 2023.

Management of BS varies by the affected body part. Treatment options include prolonged antibiotic therapy and surgical intervention. Standard antibiotic regimens for patients without focal disease include doxycycline (100 mg BID) for six weeks combined with an aminoglycoside (gentamicin 5 mg/kg/day OD for 7–10 days or streptomycin 1 g OD for 2–3 weeks) or rifampicin (600–900 mg OD) plus doxycycline (100 mg BID) for six weeks (50, 51). The Infectious Diseases Society of America (IDSA) recommends three months of antimicrobial therapy for most BS patients (32). The two most common regimens are streptomycin for 2–3 weeks with doxycycline for three months or doxycycline and rifampin, both for three months (52). The triple antibiotic regimen—rifampicin (600–900 mg OD for a minimum of 12 weeks), doxycycline (100 mg BID for a minimum of 12 weeks), and streptomycin (1 g OD for 2–3 weeks) or gentamicin (5 mg/kg/d OD for 5–7 days)—is effective and associated with lower recurrence rates (53).

Surgical management is indicated for patients with neurological symptoms due to spinal instability, spinal disruption, or epidural abscesses (54). The rise of “Instrumentation” and “Debridement” keywords indicates increased attention to surgical options. Evidence shows that spinal instrumentation is safe for patients with spinal infections (55). Traditional open surgery is recommended for patients with spinal instability, spinal cord or nerve compression, or progressive kyphosis deformity. Main surgical approaches include anterior debridement, posterior decompression (with or without instruments), and combined anterior and posterior approaches (56, 57).

Liu et al. conducted simple anterior lesion excision with internal fixation in six BS patients with cervical spine instability and cervical epidural abscess, recommending decompression and fusion for patients with combined cervical epidural abscesses (58). Yin et al. performed one-stage anterior internal fixation, debridement, and fusion in 16 lumbar brucella spondylitis (LBS) cases, concluding that this approach is safe and effective for LBS patients with spinal instability and abscess compression without posterior column involvement (59). Further studies indicate that posterior pedicle fixation, debridement, and interbody fusion are superior treatment options (18, 56, 57, 60–62). Wang et al. demonstrated that minimally invasive surgery for LBS involving portal endoscopic decompression, debridement, and interbody fusion with percutaneous screw fixation, is practical, safe, and feasible (63). The optimal surgical approach for BS remains debated and should be based on lesion location, degree of spondylolisthesis and nerve compression, and surgeon skill.

## 5 Strengths and limitations

To our knowledge, this study is the first systematic bibliometric analysis of BS. Using a comprehensive bibliometric approach, we identified significant trends, influential countries, and leading institutions. Our analysis reveals the current state of BS research, highlighting areas of active collaboration and potential gaps. These findings may provide a solid foundation for future research and policy decisions. We also realize that this study has some limitations. First, the analysis was based solely on data from the WoSCC database, potentially excluding relevant studies from other databases. Second, citation counts may be skewed by outdated studies and publication dates, affecting the perceived impact of specific research. Lastly, despite efforts to standardize the data, there may be inaccuracies in keyword extraction and incomplete content analysis due to the limitations of the analysis software. Nonetheless, these weaknesses are unlikely to affect the overall trends and conclusions of the study significantly.

## 6 Conclusion

Significant progress has been made in BS research, as evidenced by the increasing volume of publications. Current research predominantly focuses on diagnosis and complications, while management and instrumentation are emerging as potential future research frontiers. Enhancing international collaboration among researchers and institutions will be vital for driving further advancements in the field of BS.

## Data Availability

The original contributions presented in the study are included in the article/Supplementary Material, further inquiries can be directed to the corresponding author.
